# Unicystic Ameloblastoma Presenting as a Multilocular Radiolucency in the Anterior Mandible: A Case Report

**DOI:** 10.15171/joddd.2015.036

**Published:** 2015-09-16

**Authors:** Nigel R. Figueiredo, Manoj Meena, Ajit D. Dinkar, Sonam Malik, Manisha Khorate

**Affiliations:** ^1^Former Postgraduate Student, Oral Medicine and Radiology Department, Goa Dental College & Hospital, Bambolim, Goa, India; ^2^Professor & Head, Oral Medicine and Radiology Department, Goa Dental College & Hospital, Bambolim, Goa, India; ^3^Postgraduate Student, Oral Medicine and Radiology Department, Goa Dental College & Hospital, Bambolim, Goa, India; ^4^Lecturer, Oral Medicine and Radiology Department, Goa Dental College & Hospital, Bambolim, Goa, India

**Keywords:** Ameloblastoma, mandible, multilocular, unicystic

## Abstract

Ameloblastomas are tumors of odontogenic epithelial origin. The term unicystic ameloblastoma is used to describe cystic lesions with clinico-radiographic features resembling an odontogenic cyst, but histologically showing the presence of ameloblastomatous epithelium lining part of the cyst cavity. A large majority of lesions are found in the mandible, and usually cause a painless swelling of the jaws. They can be radiographically subdivided into 'dentigerous' and 'non-dentigerous' types. The unicystic ameloblastoma is believed to be less aggressive than a solid/multicystic ameloblastoma, and thus has a more favorable response to enucleation and curettage. This case report presents a case of unicystic ameloblastoma with a multilocular radiographic appearance in the anterior mandible of a 45-year-old female patient, along with a literature review of the topic.

## Introduction

An ameloblastoma is a true neoplasm of odontogenic epithelium, which is persistent and locally invasive, with aggressive but benign growth characteristics. Ameloblastomas are thought to represent 1% of all the cysts/tumors of the jaws and 18% of all the odontogenic neoplasms.^1^ There are four major clinico-radiographic types: conventional solid or multicystic ameloblastoma, which is the most common type, unicystic, peripheral and desmoplastic variants.

Conventional ameloblastomas are usually seen at 20-50 years of age, with an average age of discovery of about 40 years, and an equal sex distribution.^2^ The vast majority of ameloblastomas arise in the mandible, and the majority of these are found in the angle and ramus region.^2^

Unicystic ameloblastomas represent around 10-15% of all the intraosseous ameloblastomas.^3,4^

Over 90% of lesions are located in the mandible. Between 50% and 80% of cases are associated with an impacted tooth, most commonly a mandibular third molar, and are often called a ‘dentigerous’ variant of unicystic ameloblastoma.^2,5^ The few that are not associated with impacted teeth are called as a ‘non-dentigerous’ variant. The ‘dentigerous’ type occurs on average 8 years earlier than the ‘non-dentigerous’ variant. The mean age for unilocular, impaction-associated unicystic ameloblastomas is 22 years, whereas the mean age for the multilocular lesion unrelated to an impacted tooth is 33 years.^6^ Clinically, lesions are often asymptomatic, and usually cause a painless swelling of the jaws. On radiographic examination, as implied by the term 'unicystic', the common presentation is a unilocular radiolucency; however, occasionally a multilocular appearance may also be observed.

This paper reports a distinctive case of a unicystic ameloblastoma in a 45-year-old female patient. The lesion was not associated with an impacted tooth ('non-dentigerous' variant) and presented as a multilocular radiolucency in the anterior mandible crossing the midline which is rather unusual for these lesions.

## Case Report

**Figure 1.**
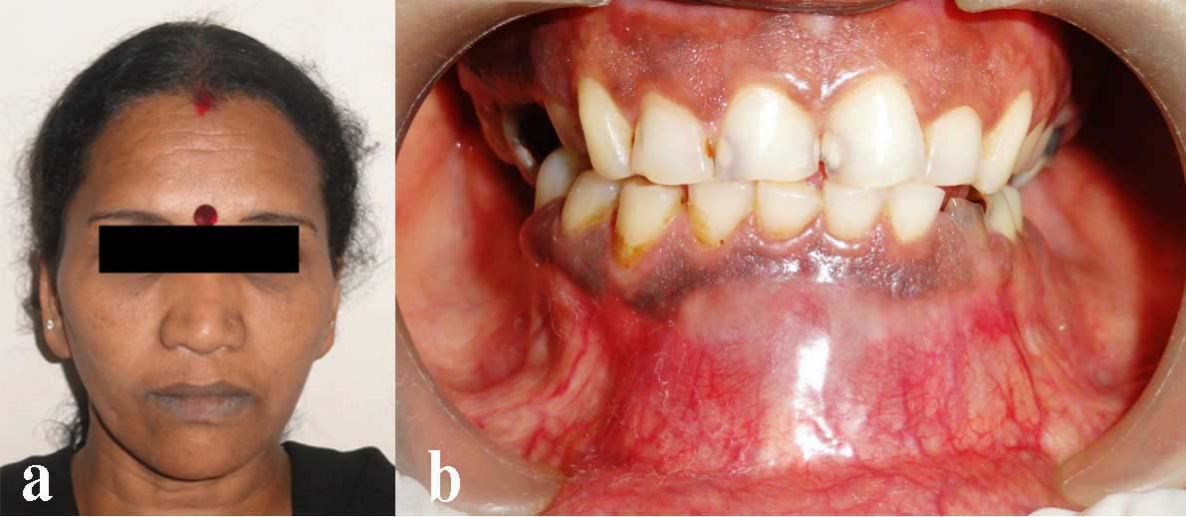
(a) Extraoral view (preoperative) showing a diffuse swelling over the chin region. (b) Preoperative intraoral view (showing swelling in the labial and buccal vestibule in the anterior mandible from the tooth #36 region crossing the midline up to the tooth #44 region).

A 45-year-old female patient reported to our outpatient department with a chief complaint of swelling in the lower anterior region for 2 months. There was no associated pain, difficulty in opening the mouth, chewing or speech. On extraoral examination, a diffuse swelling was seen over the chin region in the midline, extending from the left angle of mouth across the midline to involve the right angle of mouth area, measuring around 5×3 cm (Figure 1).

**Figure 2.**
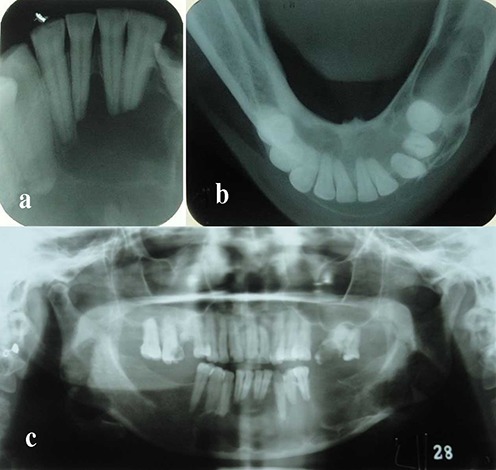
(a) Intraoral periapical radiograph showing root resorption of involved teeth and (b) mandibular true occlusal showing expansion of labial/buccal cortical plates. (c) Orthopantomogram showing a multilocular radiolucency with thin corticated borders extending from the tooth #36 region crossing the midline up to the tooth #44 region.

The overlying skin was normal with no evidence of any discharge. On palpation, the swelling was firm in consistency, tender, non-pulsatile and non-compressible, with no local rise in temperature. Intraoral examination revealed a single diffuse swelling in the mandibular labial and buccal vestibule, extending from the tooth #36 region crossing the midline up to the tooth #44 region, measuring approximately 7×3 cm, and supero-inferiorly extending from the attached gingiva to the labial and buccal vestibules (Figure 2).

The swelling was firm in consistency with a smooth surface and tender on palpation. Expansion of the lingual cortical plate was seen in relation to teeth #31, #32, #33, #34 and #41. No tenderness or mobility of the teeth in the involved area was noted. Teeth #33 and #34 were severely carious and exhibited a painful response to electric pulp testing, which lingered for a few seconds following removal of the stimulus, thus suggesting irreversible pulpitis. No significant lymphadenopathy noted.

**Figure 3.**
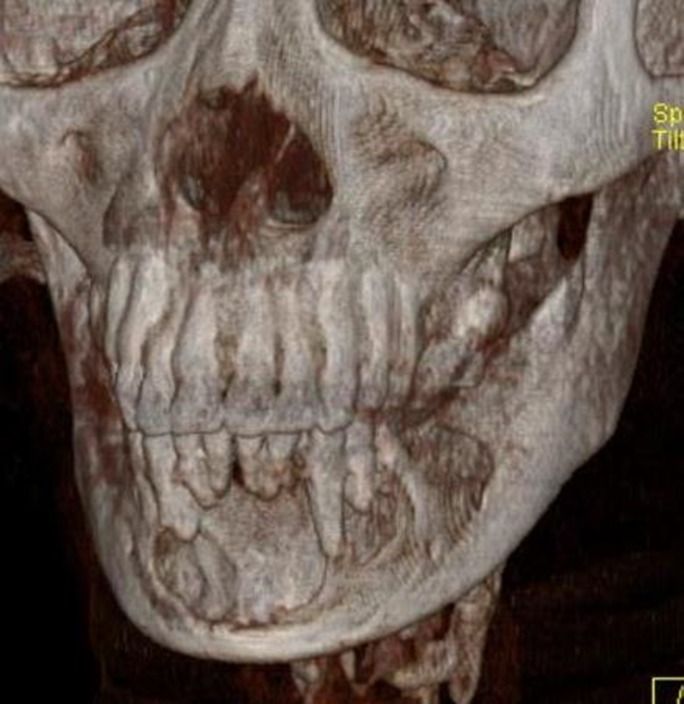
CT scan of the mandible (axial and coronal sections).

An intraoral periapical radiograph was taken, which showed root resorption of the involved teeth in the apical one-third. A mandibular true occlusal view revealed expansion of the buccal/labial cortical plate from the tooth #36 region crossing the midline up to the tooth #44 region, with the presence of a very thin corticated boundary (Figure 3).

**Figure 4.**
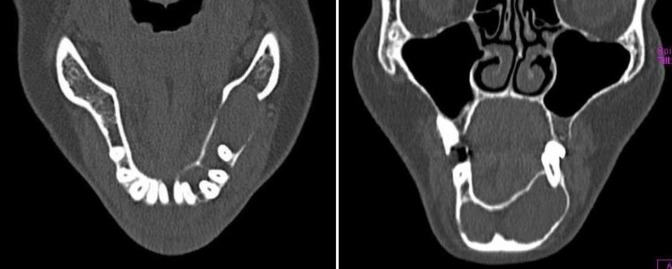
CT scan of the mandible (3D reconstruction).

Panoramic radiography showed a well-defined multilocular radiolucency with thin corticated borders extending from the left side of the mandible from the tooth #36 region, crossing the midline up to tooth #44 on the right side. The internal structure was completely radiolucent. Root resorption of teeth #31, #32, #33, #34, #35, #41, #42, #43 and #44 was noted in the apical one-third (Figure 4).

**Figure 5.**
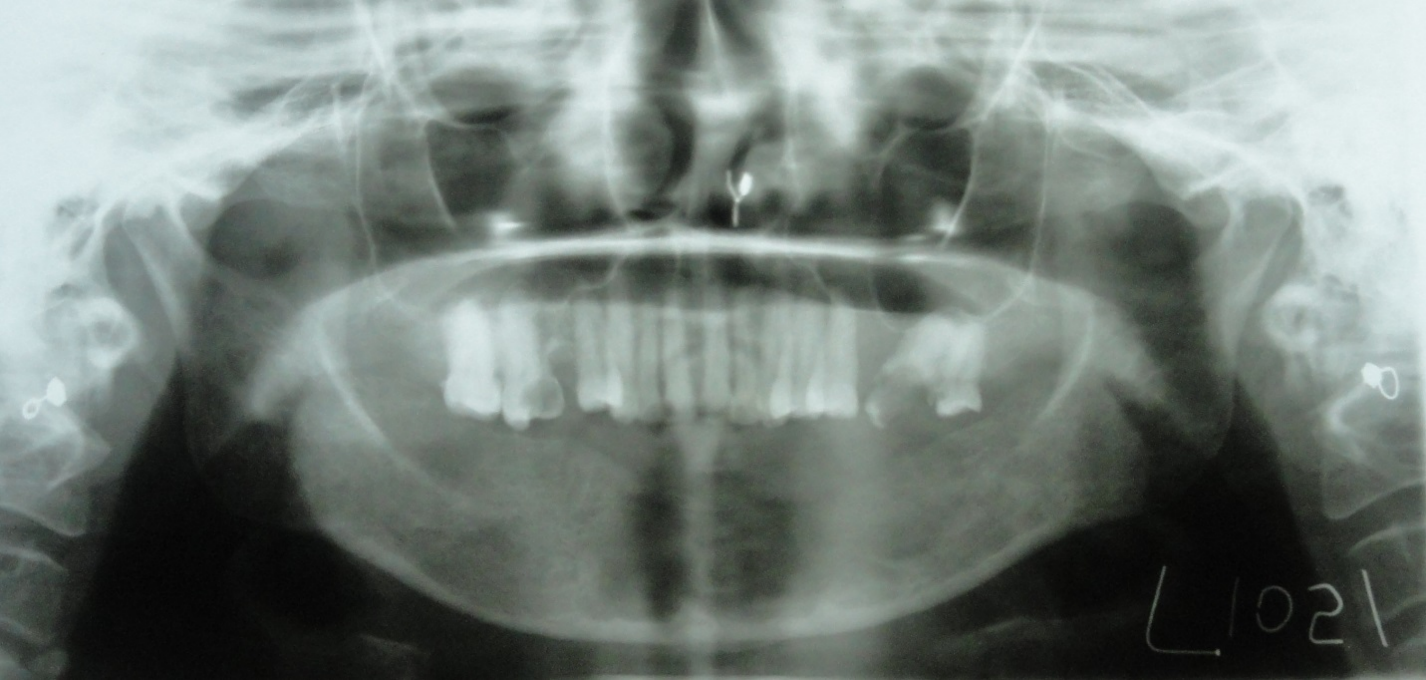
Postoperative panoramic radiograph.

**Figure 6.**
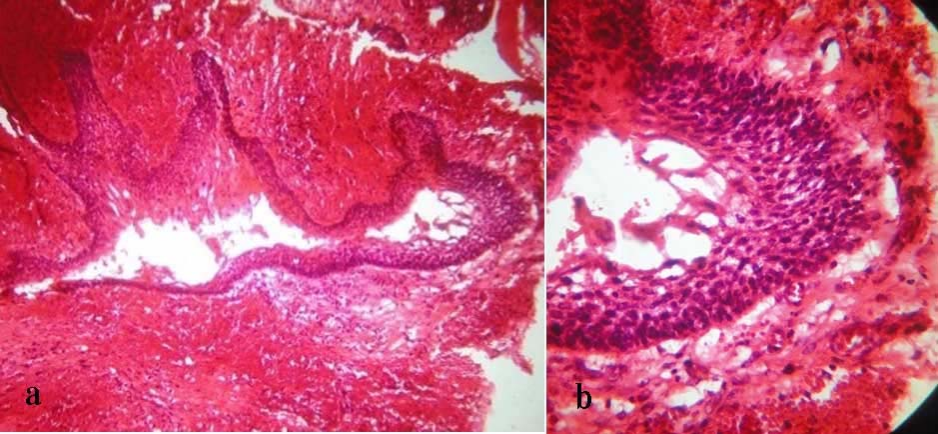
(a) Histopathology showing cystic epithelial lining with a basal cell layer of tall columnar cells with palisaded nuclei and overlying stellate reticulum-like cells in an underlying connective tissue stroma (H&E stain at ×40). (b) Histopathology of the cystic lining showed a basal cell layer composed of tall columnar cells displaying hyperchromatism and overlying stellate reticulum-like cells (H&E stain at ×100).

CT scan showed an expansile cystic lesion in the mandible, with expansion and thinning of both buccal and lingual cortices (Figures 5 and 6).

Based on the clinical and radiographic features, a provisional diagnosis of an ameloblastoma was made, with a differential diagnosis of odontogenic keratocyst, residual cyst, central giant cell granuloma and odontogenic myxoma.

An incisional biopsy was carried out, which showed a cystic lining of stratified squamous, non-keratinized epithelium, which was 2-3 cell layers thick and resembled reduced enamel epithelium, with a stroma of collagen fibers, fibroblasts, blood vessels, and areas of extravasated blood. Based on the clinico-radiographic features and findings of the incisional biopsy, the lesion was treated conservatively with careful enucleation and curettage. Histopathological examination of the enucleated tissue showed a cystic epithelial lining with an underlying connective tissue stroma. Areas of the cystic lining showed a basal cell layer composed of tall columnar cells displaying hyperchromatic, palisaded nuclei with reverse polarity and subnuclear vacuole formation. Stellate reticulum-like cells were seen overlying the basal cell layer. The ameloblastic changes were confined to the luminal surface of the cystic lining. The connective tissue stroma showed presence of collagen fibers, fibroblasts and blood vessels with a moderate-to-dense chronic inflammatory cell infiltrate. Thus, based on the histopathology of the enucleated tissue, a final diagnosis of a unicystic ameloblastoma [Ackerman's Group I] was made.

Postoperative healing was uneventful. A follow-up radiograph taken after one year showed satisfactory healing of the enucleated area. The patient has been under regular follow-up for the last 2 years, during which no recurrence has been noted.

## Discussion

The unicystic ameloblastoma was first described in 1977 by Robinson and Martinez.^4^ The term ‘unicystic ameloblastoma’ refers to those cystic lesions that show clinical and radiographic characteristics of an odontogenic cyst but on histological examination show a typical ameloblastomatous epithelium lining part of the cyst cavity, with or without luminal and/or mural tumor proliferation.^5^ Lesions may originate de novo (reduced enamel epithelium associated with a developing tooth undergoes ameloblastic proliferation with subsequent cystic development), may arise from epithelial lining of a dentigerous cyst, in which case it is called as a mural (within the wall) ameloblastoma, or may form from the cystic degeneration of solid ameloblastomas.^7^

Unicystic ameloblastomas are usually seen in younger patients (2nd-3rd decades of life) as compared to solid/multicystic ameloblastomas, with 50% occurring in the second decade of life.^2^A large majority (more than 90%) of lesions are located in the mandible, with the posterior mandible being the most commonly involved site.^5^ On clinical examination, lesions are usually asymptomatic, and patients commonly present with a painless swelling of the jaws. The patient in our case belonged to an older age group (a 45-year-old female) as compared to that described in the literature (second to third decades of life) but showed similar clinical features as reported previously. Also, our case occurred in the anterior mandible which is rather unusual, with most cases of unicystic ameloblastoma (up to 80%) thought to occur in the posterior mandible.^8^

Although unicystic ameloblastoma commonly occurs as a pericoronal radiolucency resembling a dentigerous cyst, it may also be found in the inter-radicular, periapical, or edentulous regions.^9^ Despite the fact that the term unicystic would imply a unilocular radiographic appearance, the lesion can rarely have a multilocular radiographic appearance. In the dentigerous variant, the unilocular to multilocular ratio is 4.3:1 and for the non-dentigerous type, this ratio is 1.1:1.^10^ Our case was a non-dentigerous variant and also presented as a multilocular radiolucency in the anterior mandible crossing the midline, which is rather uncommon for these lesions. The present case was also diagnosed in an older individual (45 years of age) consistent with Singh et al,^6^ who stated that the ‘non-dentigerous’ variant of unicystic ameloblastoma occurs in older individuals as compared to the ‘dentigerous’ variant.

Microscopically, the lesion appears as a well-defined, often large monocystic cavity with a lining, focally but rarely entirely, composed of odontogenic (ameloblastomatous) epithelium. It is often accompanied by an innocuous epithelium of varying histological appearance that may mimic the lining of a dentigerous or radicular cyst.^6^ Ackermann^3^ classified this entity into the following three histological groups:

Group I: Luminal (tumor confined to the luminal surface of the cyst)

Group II: Intraluminal/plexiform (nodular proliferation into the lumen without infiltration of tumor cells into the connective tissue wall)

Group III: Mural (invasive islands of ameloblastomatous epithelium in the connective tissue wall not involving the entire epithelium)

Our case showed areas of cystic lining with a basal cell layer of tall columnar cells displaying hyperchromatism, palisaded nuclei with reverse polarity and an overlying layer of stellate reticulum-like cells. The ameloblastic changes were confined to the luminal surface [Ackerman's Group I]. There was absence of any nodular proliferation into the lumen or connective tissue wall.

The epithelial lining of a unicystic ameloblastoma is not always uniformly characteristic and is often lined partly by a non-specific thin epithelium that mimics the lining of a dentigerous cyst. The true nature of the lesion may only become evident when the entire specimen is available for histological examination.^11^ This was observed in the present case, where the incisional biopsy showed features that resembled a dentigerous cyst but postoperative examination of the enucleated material revealed a unicystic ameloblastoma. Thus, a definitive diagnosis of unicystic ameloblastoma can only be reached by histological examination of the entire lesion and cannot be predicted based on only clinical or radiographic grounds. Since a preoperative incisional biopsy is not representative of the entire lesion it may result in an incorrect classification.^3^

Unicystic ameloblastoma is believed to be less aggressive, and its response to enucleation or curettage is more favorable than the classic solid or multicystic ameloblastomas.^11^ The reason for this better prognosis is that in many examples the ameloblastoma involves only the epithelial lining of the cyst or projects into its lumen.

In the present case, although the radiographic features (multilocular appearance with root resorption of the involved teeth) were suggestive of an ameloblastoma, the incisional biopsy was reported as a dentigerous cyst. However, after taking into account the location and clinico-radiographic features, besides the findings of the incisional biopsy, simple enucleation and curettage was chosen as the treatment of choice, due to low patient morbidity and minimal effect on the quality of life. Recurrence of these lesions may be long-delayed, and hence a long-term postoperative follow-up is essential to the proper management of these patients.

## Conclusion

The diagnosis of unicystic ameloblastoma based on clinical and radiographic features may be impossible in many cases due to its similarities with odontogenic cysts and tumors and because an incisional biopsy may not be able to reflect the true nature of the lesion. In the present case, we arrived at a final diagnosis of unicystic ameloblastoma only after histopathological examination of the enucleated material. Therefore, careful postoperative histopathological examination is mandatory for all the lesions, which together with a proper follow-up can play an important role in minimizing recurrences.

## Acknowledgement

Dr. Anita Spadigam, Professor & Head, and Dr. Anita Dhupar, Asst. Professor, Department of Oral and Maxillofacial Pathology, Goa Dental College & Hospital, Bambolim, Goa–INDIA, for providing the histopathological analysis, report and photographs.
